# The FENDRR/FOXC2 Axis Contributes to Multidrug Resistance in Gastric Cancer and Correlates With Poor Prognosis

**DOI:** 10.3389/fonc.2021.634579

**Published:** 2021-03-22

**Authors:** Hao Liu, Zhe Zhang, Yanan Han, Ahui Fan, Haiming Liu, Xiangyuan Zhang, Yanhong Liu, Rugang Zhang, Wanning Liu, Yuanyuan Lu, Daiming Fan, Xiaodi Zhao, Yongzhan Nie

**Affiliations:** ^1^ State Key Laboratory of Cancer Biology and National Clinical Research Center for Digestive Diseases, Xijing Hospital of Digestive Diseases, Fourth Military Medical University, Xi’an, China; ^2^ Department of Gastroenterology and Hepatology, Hainan Branch of Chinese PLA General Hospital, Sanya, China; ^3^ Department of Gastroenterology, Xi’an Children’s Hospital, Xi’an, China; ^4^ School of Software Engineering, Beijing Jiaotong University, Beijing, China; ^5^ Department of Gastroenterology and Hepatology, 952 Hospital of the Chinese PLA Ground Force, Golmud, China; ^6^ Department of Traditional Chinese Medicine Physical Therapy and Rehabilitation, Seventy-Fourth Army of the PLA Hospital, Guangzhou, China; ^7^ College of Life Sciences, Northwest University, Xi’an, China

**Keywords:** gastric cancer, multidrug resistance, FENDRR, FOXC2, miR-4700-3p

## Abstract

The dysregulation of long non-coding RNAs (lncRNAs) and transcription factors (TFs) is closely related to the development and progression of drug resistance in cancer chemotherapy. However, their regulatory interactions in the multidrug resistance (MDR) of gastric cancer (GC) has largely remained unknown. In this study, we report a novel oncogenic role of lncRNA FENDRR in conferring MDR in GC by coordinated regulation of FOXC2 expression at the transcriptional and posttranscriptional levels. *In vitro* and *in vivo* experiments demonstrated that downregulation of FENDRR expression remarkably decreased drug resistant ability of GC MDR cells while upregulation of FENDRR expression produced the opposite effect. FENDRR overexpression was observed in MDR GC cell lines, patient-derived xenografts, and clinical samples. And the high levels of FENDRR expression were correlated with poor prognosis in GC patients. Regarding the mechanism, FENDRR was revealed to increase proto-oncogene FOXC2 transcription by performing an enhancer-like role in the nucleus and by sponging miR-4700-3p in the cytoplasm. Both FOXC2 and miR-4700-3p were shown to be functionally involved in the FENDRR-induced chemoresistance. In addition, there is a positive correlation between FENDRR and FOXC2 expression in clinic and the overexpressed FOXC2 indicated a poor prognosis in GC patients. Collectively, our findings provide a new perspective for the lncRNA-TF regulatory interaction involved in MDR, suggesting that targeting the FENDRR/FOXC2 axis may be an effective approach to circumvent GC chemoresistance.

## Introduction

Gastric cancer (GC), which is the fourth most common cancer worldwide, remains an enormous threat to human health despite the decreases in incidence and mortality observed in recent years (1). Because most patients with GC are diagnosed at an advanced stage, when surgery is impracticable, chemotherapy plays a pivotal role in the treatment of GC (2). However, cancer cells can develop resistance to various chemotherapeutic drugs, a trait known as multidrug resistance (MDR), which serves as a major impediment to successful chemotherapy (3). Therefore, an understanding of the pathogenetic and regulatory mechanisms of MDR in GC is urgently needed, which may provide potential intervention targets to overcome MDR.

Transcription factors (TFs) are key regulators of gene expression, the dysregulation of which frequently occurs and has been shown to be an important contributor to MDR (4). The Forkhead/winged helix-box (FOX) family is an evolutionarily conserved group of multifunctional TFs that function by binding a conserved DNA sequence termed the “forkhead box” (5). Recent studies have demonstrated that dysregulated FOX members are closely associated with cancer drug resistance (6). FOXO3a has been shown to antagonize the cytotoxic effects of cisplatin (CDDP) and doxorubicin in colon cancer and chronic myeloid leukemia, respectively (7, 8). FOXF1 was previously shown to promote angiogenesis and enhance bevacizumab resistance by inducing the transcription of VEGFA in colon cancer (9). As a member of the C family of FOX proteins, FOXC2 has been reported to play crucial roles in cancer progression, including in chemoresistance. Furthermore, FOXC2 can promote epithelial to mesenchymal transition (EMT) in basal-like breast cancer and non-small cell lung cancer, which is an important mechanism in the induction of metastasis and drug resistance (10, 11). In addition, FOXC2 was shown to confer resistance to CDDP and fluorouracil (5-FU) in ovarian cancer and colorectal cancer, respectively (12, 13). Although FOXC2 has been observed to be upregulated in GC (14), suggesting its oncogenic roles in gastric carcinogenesis, its functions in GC MDR have yet to be investigated.

Long non-coding RNAs (lncRNAs), a class of non-protein-coding transcripts that are greater than 200 bases in length, also play crucial roles in gene regulation (15). Recently, lncRNAs have received attention as important regulators in cancer drug resistance through multifarious mechanisms (16, 17). In the nucleus, lncRNAs can regulate the expression of drug resistance-related genes in either a *cis* (on neighboring genes) or *trans* (on distantly located genes) manner. For instance, lncHOXA10 promotes the expression of its nearby gene HOXA10 in *cis* by recruiting the SNF2L chromatin remodeling complex to its promoter in liver cancer (18). LINC00968 attenuates drug resistance in breast cancer by directly binding HEY1 to inhibit WNT2 expression in *trans* (19). In the cytoplasm, lncRNAs can act as competing endogenous RNAs (ceRNAs) to regulate genes involved in chemoresistance. For example, TINCR can target miR-125b in breast cancer cells, thereby increasing HER-2 expression and inducing trastuzumab resistance (20). NR2F1-AS1 sponging of miR-363 promotes oxaliplatin resistance in HCC by increasing ABCC1 expression (21). FOXF1 adjacent non-coding developmental regulatory RNA (FENDRR), also known as lncRNA FOXF1-AS1, is aberrantly expressed in various types of cancer, such as breast cancer, lung cancer, and chronic myelogenous leukemia (22–24). Although the results of most studies suggest that FENDRR functions as tumor suppressor, the roles of FENDRR in cancers is complicated or even contradictory in similar malignant phenotypes. For instance, a previous study demonstrated that FENDRR can promote the migration and invasion of osteosarcoma cells (25), whereas another study reported that FENDRR was able to repress migration and invasion in cholangiocarcinoma (26). These discrepancies suggest that the role of FENDRR may be tumor stage- or cell context-dependent, and the function and underlying mechanisms of FENDRR in drug resistance of GC remain to be elucidated.

In this study, we observed that FENDRR expression was significantly upregulated in GC MDR cell lines, patient-derived xenografts (PDXs), and clinical tissue samples. We demonstrated that FENDRR promoted GC MDR both *in vitro* and *in vivo* and that FENDRR can increase the expression of the drug resistance-promoting molecule FOXC2 by performing an enhancer-like function and sponging miR-4700-3p. Collectively, our results provide novel insights into the FENDRR/miR-4700-3p/FOXC2 regulatory axis involved in MDR and suggest a crucial nexus between lncRNA and TF in GC progression.

## Materials and Methods

### Cell Culture

The human GC cell line SGC7901 was previously obtained from the Academy of Military Medical Science and is preserved in the State Key Laboratory of Cancer Biology, China. The multidrug-resistant sublines SGC7901/ADR and SGC7901/VCR were established in our laboratory as previously described (27). SGC7901, SGC7901/ADR, and SGC7901/VCR cells were cultured in RPMI 1640 medium (Thermo Fisher Scientific Gibco, Beijing, China) supplemented with 10% bovine growth serum (Gibco BRL), 100 U/ml penicillin, and 100 µg/ml streptomycin (HyClone) under an atmosphere with 5% CO_2_ at 37°C. ADR (Sigma, Inc., St. Louis, MO, USA; 0.5 µg/ml) and VCR (Sigma, 1.0 µg/ml) were added to the culture medium of the SGC7901/ADR and SGC7901/VCR cell lines, respectively, to maintain their drug-resistant phenotypes.

### LncRNA Microarray Profiling

RNA was extracted from SGC7901, SGC7901/ADR, and SGC7901/VCR cells using a RNeasy Plus Mini kit (Qiagen, Hilden, Germany). lncRNAs were sequenced, and microarray analysis was performed using an Agilent Human lncRNA microarray V.2.0 platform (GPL18109) as previously described (20).

### Tissue Microarray, FISH, and IHC

The GC tissue microarray containing 80 cases of GC and paired adjacent non-cancerous tissues was obtained from Alenabio (Xi**’**an, China). All the samples were shown to be correctly labeled clinically and pathologically.

FENDRR expression in GC cells and tissue samples was detected with FISH assays as previously described (28). Briefly, a double digoxigenin (DIG)-labeled probe against FENDRR was synthesized (Exiqon). An anti-DIG HRP-conjugated antibody (PerkinElmer) and tyramide signal amplification (TSA) Cy3 (PerkinElmer) were used, and Hoechst 33342 was used to stain nuclei. Slides were imaged with a Nikon ECLIPSE Ti confocal microscope, and the staining intensity was assessed as previously described (29).

IHC for FOXC2 was performed on sections of tumor tissue samples from nude mouse xenografts and GC patients as previously described (30). Briefly, tissue sections were deparaffinized, subjected to antigen retrieval and endogenous peroxidase inactivation, and incubated with primary antibodies against FOXC2 (R&D Systems, AF6989) and Ki-67 (Abcam, ab15580). Then, the sections were incubated with a peroxidase-conjugated secondary antibody (Santa Cruz), which was followed by visualization with diaminobenzidine and image acquisition with a light microscope (Olympus, Japan). The final immunoactivity scores of each section were determined by two independent observers in a blinded manner according to standard procedures described previously (30). IHC scores of 0–12 were calculated and graded as negative (−, score: 0), weak (+, score: 1–4), moderate (+ +, score: 5–8) or strong (+ + +, score: 9–12). Samples with IHC scores **>**4 were considered to have high expression, while samples with IHC scores **≤**4 were considered to have low expression.

### RNA Extraction, cDNA Synthesis, and Real-Time PCR

Total RNA was extracted from cultured cells and GC xenografts using a RNeasy Plus Mini kit (Qiagen, Hilden, Germany), and miRNA was extracted using an miRNeasy Mini kit (Qiagen) according to the manufacturer**’**s instructions. Then, cDNA was synthesized using a PrimeScript RT reagent kit (TaKaRa, Dalian, China), and SYBR Premix Ex Taq II (TaKaRa) was used to amplify the double-stranded cDNA of interest. qPCR primers for miR-4700-3p and U6 were purchased from RiBoBio (Guangzhou, China), while those for FENDRR, FOXC2, ABCB1, and β-actin (ACTB) were synthesized by TaKaRa (Dalian, China). The levels of U6 and ACTB were used as internal controls for miRNA and mRNA levels, respectively. The 2^-ΔΔCt^ method was used to calculate relative RNA expression. The primer sequences are listed in **Supplementary Table S1**.

### Protein Extraction and Western Blot Analysis

Total protein was prepared from GC cells or tissues using RIPA buffer (Beyotime, Shanghai, China) with a complete protease inhibitor cocktail (Roche, Manheim, Germany). Approximately 20–50 µg of denatured protein was fractionated by SDS-PAGE and transferred to nitrocellulose membranes. The membranes were blocked and then incubated with anti-FOXC2 (Abcam, ab5060), anti-PARP (Cell Signaling Technology (CST), #9532), anti-cPARP (CST, #9541), and anti-ACTB (Sigma-Aldrich, A1978) antibodies overnight at 4°C. Proteins were visualized using Dura SuperSignal Substrate (Pierce, USA), and the blots were scanned using a Molecular Imager ChemiDox XRS+ Imaging System with Image Lab software (Bio-Rad Laboratories).

### Colony Formation Assay

Transfected or infected cells (0.5 × 10^3^ cells per well) were seeded in a six-well plate, which was followed by the addition of ADR (6 µg/ml) into the culture medium on the second day. After 2 weeks, visible colonies were fixed with 10% formaldehyde for 5 min and stained with 0.5% crystal violet for 5 min. Then, the number of colonies was counted using ImageJ.

### CCK-8 Assay

Cell viability was determined by CCK-8 analysis as described previously (24). Briefly, 5 × 10^3^ cells were seeded in a 96-well plate. After incubating for 24 h, the cells were treated with varying concentrations of ADR or 5-FU, incubated for 72 h, and then incubated with a CCK-8 solution (Dojindo, Kumanoto, Japan) for 3 h. The absorbance at 450 nm was measured with a Varioskan**^®^** Flash spectrometer (Thermo Fisher Scientific, Waltham, USA).

### Apoptosis Assay

The apoptosis rates of cells treated with 5-FU (0.5 µg/ml for SGC7901 cells and 15 µg/ml for SGC7901/ADR and SGC7901/VCR cells) for 24 h were measured by flow cytometry as previously described (31). Briefly, cells were washed and resuspended in 100 µl of binding buffer at a concentration of 1 × 10^6^/ml. Then, 5 µl of annexin V-fluorescein isothiocyanate (FITC; Pharmingen, San Diego, CA, USA) and 10 µl of 20 µg/ml propidium iodide (Sigma) were added to the cells. After incubating at room temperature for 15 min, 400 µl of annexin-binding buffer was added to each sample, and the samples were maintained on ice until the stained cells were analyzed with a flow cytometer (FACScan, BD Biosciences, USA).

### *In Vivo* Drug Sensitivity Assay

Approximately 1 × 10^7^ SGC7901/ADR cells infected with lentiviral shFENDRR, shFOXC2, or the corresponding negative control were subcutaneously injected into both flanks of female BALB/c nude mice (n = 5). One week later, the mice were intraperitoneally injected with PBS containing ADR (30 mg/m^2^) once per week. On days 7, 14, 21, and 28, Tumor volume was calculated using the formula: tumor maximum diameter (L) × the right-angle diameter to that axis (W)^2^/2. On day 28, the mice were humanely sacrificed, and the tumors were weighed and imaged. Total RNA of xenograft was extracted using TRIzol reagent as previously described (32). Paraffin sections were prepared, and IHC was performed with staining for Ki-67 and FOXC2. The protocol for the animal studies was approved by the Fourth Military Medical University Animal Care Committee.

### Constructs, Oligonucleotides, and Cell Infection and Transfection

miR-4700-3p mimics, inhibitors, and negative control were chemically synthesized and purified by RiBoBio (Guangzhou, China). siRNAs targeting FENDRR and FOXC2 were purchased from GenePharma (Shanghai, China). The FENDRR expression vector was generated by RT-PCR amplification of FENDRR cDNA, which was then subcloned into the vector pcDNA 3.1 (Invitrogen). Expression vectors encoding FOXC2 were constructed by cloning the open reading frames and downstream 3**’**-untranslated region (UTR) into the vector pcDNA 3.1. The shRNA sequences of FENDRR and FOXC2 were amplified and cloned into the pEZX-MR03 lentiviral transfer vector (GeneCopoeia). Oligonucleotide transfection was performed using Lipofectamine RNAiMAX Transfection Reagent (Thermo Fisher Scientific) following the manufacturer**’**s instructions. The FENDRR and FOXC2 expression plasmids were transfected into the indicated cells using Lipofectamine 2000 (Thermo Fisher Scientific). The sense-strand sequences of the FENDRR-specific siRNAs designed to target human cells were as follows: FENDRR siRNA#1, 5**’**-CCGAAGAUACCAAGUGAAATT-3**’**; FENDRR siRNA#2, 5**’**-CAGAAAACAUCGGAUUUACTT-3**’**; and FENDRR siRNA#3, 5**’**-GGAGGGAAUUAGAAGCGUUTT-3**’**. The FOXC2 shRNA targeting sequence was 5**’**-CCACACGTTTGCAACCCAA-3**’**. Successful knockdown of FENDRR by siRNA transfection was confirmed by PCR. Cells with stably downregulated FENDRR and FOXC2 expression were established by transduction with lentiviruses expressing FENDRR and FOXC2 shRNA, respectively. Cells infected with lentivirus were selected with puromycin (2 µg/ml; Sigma) for 2 weeks.

### Establishment of PDXs

This study was approved by the Hospital**’**s Protection of Human Subjects Committee, and the protocol for the animal studies was approved by the Fourth Military Medical University Animal Care Committee. All patients provided informed consent. Fresh GC tissue samples were obtained from patients who underwent surgical resection at the Xijing Hospital of Digestive Diseases. The tumor tissue samples were collected, sliced into 1–3 mm^3^ fragments and subcutaneously implanted in the dorsal flanks of 6-week-old NSG nude mice. Tumor volume was assessed by bilateral caliper measurements and calculated twice per week after implantation. Once the xenografts reached 100 mm^3^, the mice were then randomly divided into four groups and treated with either PBS containing ADR, 5-FU, and CDDP or saline injected intraperitoneally three times per week for 3 weeks. Chemotherapy responsiveness was assessed based on the ratio of the average volume in the treatment group to that in the control group. A classification of **“**chemotherapy responsive**”** (ratio **<**0.42) or **“**chemotherapy resistant**”** (ratio **>**0.42) was assigned to each mouse in the drug-treated group (33). RNA was extracted from the xenografts, and FENDRR or ABCB1 expression was detected in the drug-treated and control groups.

### Luciferase Reporter Assay

Luciferase reporter assays were performed as described previously (32). Briefly, mutations of miR-4700-3p binding site were generated by QuickChange site-directed mutagenesis. The full-length sequence of FENDRR and the 3**’**-UTR of FOXC2 containing wild-type or mutant miR-4700-3p binding sites were subcloned into the vector psiCHECK-2 (Promega). HEK293 cells were co-transfected with these wild-type or mutant plasmids in addition to the miR-4700-3p mimic or negative control (at a final concentration of 50 nmol/l). For luciferase reporter assays used to measure FOXC2 promoter activity, the indicated cells were co-transfected with the pGL3-FOXC2 promoter fragment, pRL-SV40 Renilla luciferase reporter, and pcDNA3.1-FENDRR, pcDNA3.1-shFENDRR, or an empty vector control. At 48 h after transfection, firefly and Renilla luciferase activities were measured using a dual-luciferase assay kit (GeneCopoeia). Firefly luciferase activity was normalized to Renilla luciferase activity for each transfected well. Three independent experiments were performed in triplicate.

### Kaplan-Meier Plotter Analysis

The prognostic value of the FENDRR and FOXC2 genes in GC was analyzed using Kaplan-Meier plotter (http://kmplot.com/analysis/), an online database that can assess the effects of genes on survival using cancer samples (34). This database includes 1,065 GC patients with a mean follow-up time of 33 months. Patients with higher or lower expression of FENDRR or FOXC2 (Probe ID: 243059_at for FENDRR and 214520_at for FOXC2) were segregated and analyzed using the log-rank test. Hazard ratios with the corresponding 95% confidence intervals and log-rank *P* values were noted.

### Datasets

TCGA (https://cancergenome.nih.gov) datasets were used to determine the correlation between FENDRR and FOXC2 expression with miR-4700-3p expression in human gastric cancer specimens and prognostic value of miR-4700-3p.

### Statistical Analysis

Normally distributed data are presented as the means ± SD. All analyses were performed using SPSS (version 22.0). Student**’**s t-test (two-tailed), ANOVA (Dunnett**’**s or LSD *post hoc* **** test), Pearson correlation coefficients or χ^2^-tests were used to analyze data according to the type of experiment. *P* < 0.05 was considered significant.

## Results

### FENDRR Expression Is Upregulated in Multidrug-Resistant GC Cells and Correlates With Poor Prognosis in GC Patients

**Figure 1:**
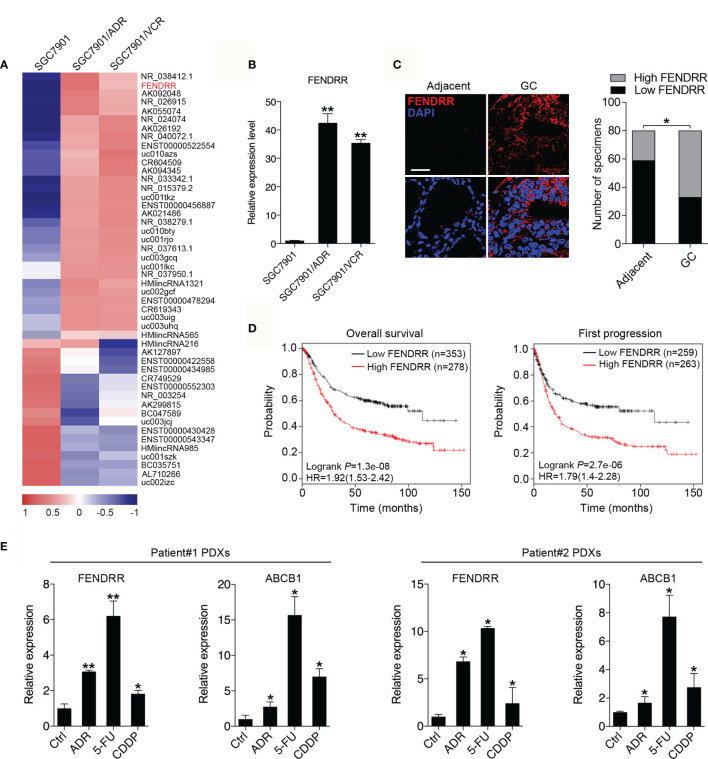
Upregulation of FENDRR expression in GC tissues and multidrug-resistant cells indicates a poor prognosis **(A)** Heat map showing the lncRNAs with significantly increased or decreased expression in the multidrug-resistant GC cell lines SGC7901/ADR and SGC7901/VCR compared with the parental SGC7901 cells, as determined using an Agilent Human lncRNA microarray. The scale bar shows color-coded differences in expression from the mean. **(B)** qRT-PCR results showing the upregulation of FENDRR expression in SGC7901/ADR and SGC7901/VCR cells compared with SGC7901 cells. **(C)** Representative images of FISH assays for FENDRR (red) in 80 paired GC and adjacent normal tissue samples are shown. Scale bar, 50 μm. **(D)** Kaplan-Meier analysis was used to evaluate the correlation between FENDRR expression and overall (left) and first progression (right) survival in patients with GC. **(E)** qRT-PCR was used to analyze FENDRR and ABCB1 expression in two drug resistant patient-derived xenografts (PDXs). **P < 0.01, *P < 0.05. Data represent mean ± s.d. in **(B, C, E)**.

To identify lncRNAs that may contribute to the MDR phenotype of GC, we performed genome-wide lncRNA microarray profiling of SGC7901 cells and their MDR derivatives SGC7901/ADR and SGC7901/VCR cells, which were derived from SGC7901 by stepwise selection using adriamycin (ADR) and vincristine (VCR) as inducing reagents, respectively. We observed that 31 lncRNAs were significantly increased, while 17 lncRNAs were significantly decreased, in both SGC7901/ADR and SGC7901/VCR cells (Log10 FC>1, P < 0.01; **Figure 1A**). Among these lncRNAs, FENDRR was particularly interesting owing to it being the most significantly upregulated, with 31.8- and 17.3-fold increases in expression observed in SGC7901/ADR and SGC7901/VCR cells, respectively. The upregulation of FENDRR was confirmed by qPCR in both MDR cell lines (**Figure 1B**). Moreover, FENDRR expression was examined by fluorescence *in situ* hybridization (FISH) in a tissue microarray containing 80 pairs of GC and adjacent nontumor tissues. We observed that FENDRR expression was significantly increased in GC tissues compared with their matched adjacent non-tumor tissues (**Figure 1C**). Further correlation analyses revealed that higher FENDRR expression in tumors was significantly associated with more aggressive tumor phenotypes in GC patients (**Table 1**). The prognostic value of FENDRR was investigated using the Kaplan-Meier plotter database (34), and the results showed that GC patients with higher FENDRR expression exhibited significantly shorter overall and first progression survival times than those with lower FENDRR expression (**Figure 1D**). We further assessed FENDRR expression in mice bearing PDXs that were treated with ADR, 5-FU, or CDDP. In two groups, FENDRR expression levels were significantly increased in PDXs that received chemotherapy treatment compared with saline-treated PDXs (**Figure 1E**), along with upregulation of ABCB1, a well-known MDR protein (35). These results suggest that FENDRR is involved GC progression and may promote the development of MDR.

**Table 1:** Correlation of FENDRR expression and patients’ clinicopathological variables in GC tissues.

Clinicopathological variables		Tumor FENDRR expression			*P* Value
		All cases (n = 80)	Low (n = 33)	High (n = 47)	
Age (years)	<50	28	10	18	0.486
	≥50	52	23	29	
Gender	Female	37	16	21	0.821
	Male	43	17	26	
Tumor size	<5 cm	35	20	15	0.013
	≥5 cm	45	13	32	
Grade of differentiation	G1	19	12	7	0.041
	G2	31	13	18	
	G3	30	8	22	
Tumor invasion	T1	13	9	4	0.029
	T2	18	10	8	
	T3	29	8	21	
	T4 a/b	20	6	14	
Lymph node metastasis	Absent	34	19	15	0.038
	Present	46	14	32	
Distant metastasis	M0	31	18	13	0.02
	M1	49	15	34	

### FENDRR Is Important for Maintaining Chemoresistance in MDR Cells *In Vitro* and *In Vivo*

**Figure 2:**
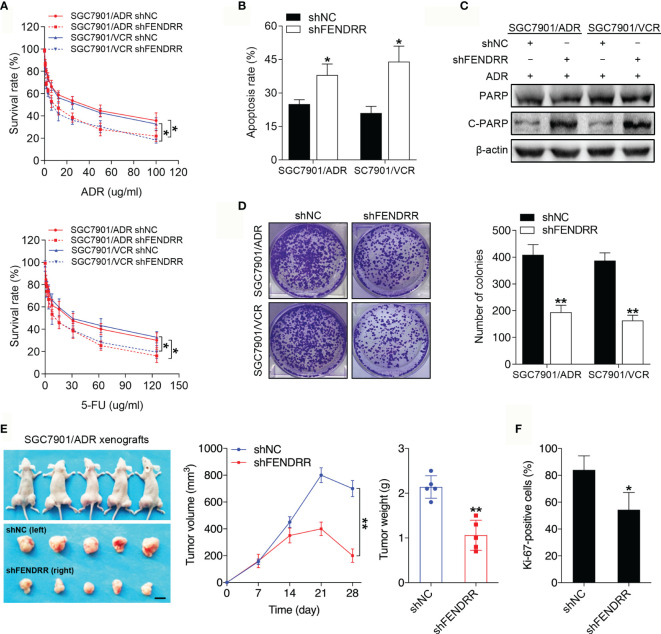
FENDRR regulates the sensitivity of GC cells to chemotherapeutic agents *in vitro* and *in vivo* **(A)** Survival of indicated cells after step-up concentration of ADR and 5-FU treatment for 72 h was evaluated using the CCK-8 assay. **(B)** Apoptotic rate of the indicated cells treated with 5-FU. **(C)** Western blot analysis of PARP and C-PARP expression in the indicated cells treated with ADR. **(D)** Colony formation assay with the indicated cells treated with ADR. **(E)** LV-shFENDRR-infected cells were transplanted into the right flank of nude mice, and the corresponding control cells were transplanted into the left side. Left, representative images of tumors formed in nude mice (n=5) after ADR treatment are shown, scale bar, 1 cm. Middle, the tumor volumes of different groups were measured at the indicated time points. Right, the tumor weights of different groups measured after tumor isolation. **(F)** Quantification of the Ki67-positive staining of cells in xenografts from nude mice. **P < 0.01, *P < 0.05, data are representative of three independent experiments, error bars, s.d.

To investigate the roles of FENDRR in GC chemoresistance, we silenced FENDRR expression with three siRNAs targeting FENDRR in SGC7901/ADR and SGC7901/VCR cells and established stable cell lines infected with FENDRR shRNA lentivirus (shFENDRR) based on the most efficient siRNA sequence (**Supplementary Figure 1A**). We observed that downregulation of FENDRR in SGC7901/ADR and SGC7901/VCR cells attenuated cell viability in the presence of ADR and 5-FU (**Figure 2A**). In addition, downregulation of FENDRR enhanced apoptosis and led to increased expression of cleaved PARP, a marker of cells undergoing apoptosis (36), in MDR cells treated with ADR (**Figures 2B, C** and **Supplementary Figure 1B**). Moreover, FENDRR knockdown impaired the colony-formation potential of MDR cells in receiving chemotherapy (**Figure 2D**). To determine whether FENDRR regulates chemoresistance *in vivo*, we transplanted SGC7901/ADR cells that had been stably transduced with shFENDRR or a negative control into nude mice (**Supplementary Figure 1C**). The volumes and weights of tumors in the shFENDRR-transfected group after ADR treatment were markedly decreased compared to those observed in the negative control group (**Figure 2E**). Immunohistochemical (IHC) staining results showed that FENDRR downregulation suppressed proliferative activity in the shFENDRR transfected tumors, as indicated by the percentage of cells staining positive for Ki-67 (**Figure 2F** and **Supplementary Figure 1D**). These observations indicated that FENDRR downregulation could sensitize MDR GC cells to chemotherapy.

### FENDRR Promotes Drug Resistance by Performing an Enhancer-Like Role to Facilitate FOXC2 Expression in GC

**Figure 3:**
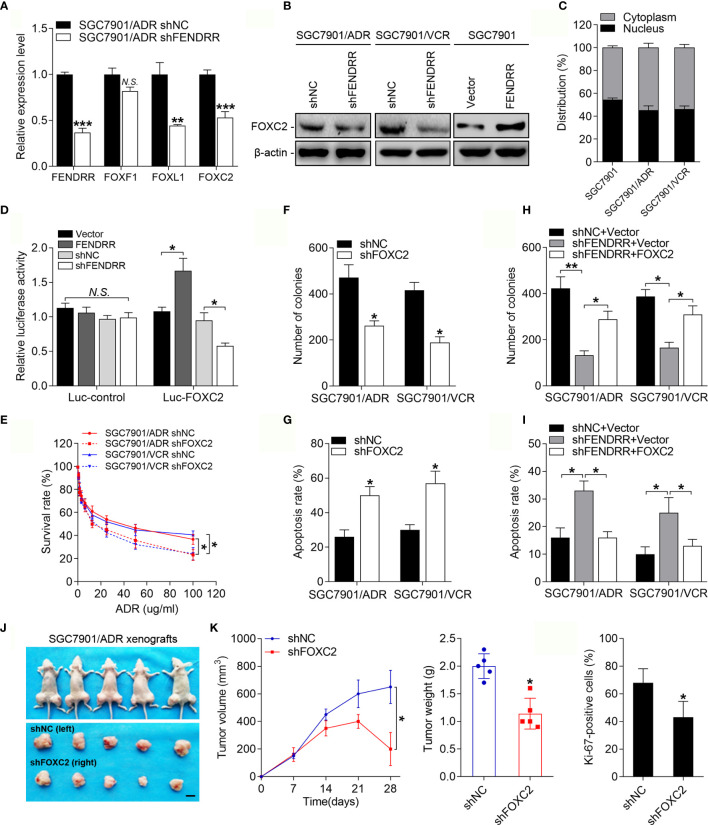
FENDRR-regulated FOXC2 overexpression promotes GC drug resistance **(A)** qRT-PCR analysis of FENDRR, FOXF1, FOXL1 and FOXC2 expression in SGC7901/ADR cells infected with FENDRR shRNA or negative controls. **(B)** Western blot analysis of FOXC2 expression in SGC7901/ADR and SGC7901/VCR cells infected with FENDRR shRNA and in SGC7901 cells transfected with FENDRR vectors and the corresponding negative controls. **(C)** Expression levels of FENDRR in the nucleus and cytoplasm of the indicated cells was measured by qPCR. U1 and GAPDH were used as nuclear and cytoplasmic controls, respectively. **(D)** HEK-293 cells were transfected with FOXC2 luciferase reporter constructs and FENDRR-expressing vectors or shRNA and the corresponding negative controls. Luciferase activity values were measured and analyzed. Luciferase values are normalized to empty vector control values. **(E)** Survival of indicated cells after step-up concentration of ADR treatment for 72 h was evaluated using the CCK-8 assay. **(F, G)** Colony formation assay with the indicated cells treated with ADR **(F)** and the apoptotic rate of the indicated cells treated with 5-FU **(G)** are shown. **(H, I)** Colony formation assay **(H)** and apoptosis assay **(I)** of SGC7901/ADR and SGC7901/VCR cells infected with LV-shFENDRR with FOXC2 vector or control vector after ADR and 5-FU treatment, respectively. **(J)** LV-shFOXC2-infected cells were transplanted into the right flank of nude mice, and the corresponding control cells were transplanted into the left side. Left, representative images of tumors formed in nude mice (n=5) after ADR treatment are shown, scale bar, 1 cm. Middle, the tumor volumes of different groups were measured at the indicated time points. Right, the tumor weights of different groups measured after tumor isolation. **(K)** Quantification of the Ki67-positive staining of cells in xenografts from nude mice. **P < 0.01, *P < 0.05, *N.S.*, not significant (*P* > 0.05), error bars, s.d.

FENDRR is located on chromosome 16 (16q24.1), which harbors several members of the FOX TF family, including FOXF1, FOXC2, and FOXL1 in an approximately 60-kb region (**Supplementary Figure 2A**). Since lncRNAs can play enhancer-like roles to promote the expression of neighboring protein-encoding genes (37), we assessed whether FENDRR functions by regulating specific FOX family members known to play important roles in cancer drug resistance (6). The expression levels of FOXF1, FOXC2, and FOXL1 were upregulated in both MDR cell lines (**Supplementary Figure 2B**), while inhibition of FENDRR reduced the mRNA levels of FOXC2 and FOXL1 but not FOXF1 in these cells (**Figure 3A** and **Supplementary Figure 2C**). Considering that FOXL1 has been extensively studied as a tumor suppressor (38), we selected FOXC2, a recognized oncogene in multiple cancer types (39), for further investigation. Consistently, silencing FENDRR suppressed FOXC2 protein expression in MDR cells, while overexpressing FENDRR increased FOXC2 expression in SGC7901 cells (**Figure 3B**). We then assessed the intracellular distribution of FENDRR and observed substantial FENDRR in the nuclei of the MDR cells (**Figure 3C** and **Supplementary Figure 2D**), which is a prerequisite for FENDRR to play an enhancer-like role. We subsequently performed dual-luciferase reporter assays to evaluate if FENDRR increased FOXC2 expression at the transcriptional level. FENDRR overexpression significantly increased luciferase activity of the reporter construct, while FENDRR knockdown resulted in partial loss of transcriptional activity (**Figure 3D**). These data suggest that FENDRR plays an enhancer-like role in the regulation of FOXC2 expression in GC cells. We next investigated whether FOXC2 is functionally involved in the FENDRR-mediated chemoresistance. Downregulation of FOXC2 expression decreased the survival rate of SGC7901/ADR and SGC7901/VCR cells after ADR and 5-FU treatment (**Figure 3E** and **Supplementary Figure 2E**). Furthermore, FOXC2 downregulation also resulted in decreased cell proliferation and an increased percentage of apoptotic MDR cells treated with ADR and 5-FU (**Figures 3F, G** and **Supplementary Figure 2F**). We then investigated whether FOXC2 is responsible for FENDRR-mediated chemoresistance in GC cells. To this end, we transduced MDR cells with FENDRR shRNA and then transfected with a FOXC2 construct or control vector (**Supplementary Figure 2G**). Colony formation and apoptosis assay results demonstrated that FOXC2 overexpression partially rescued the impaired proliferative and anti-apoptotic abilities of MDR cells caused by FENDRR downregulation (**Figures 3H, I** and **Supplementary Figure 2I**). These results suggest that FENDRR promotes GC MDR in a FOXC2-dependent manner. To assess whether FOXC2 promotes drug resistance in GC cells *in vivo*, we transplanted SGC7901/ADR cells stably expressing shFOXC2 or a negative control into nude mice (**Supplementary Figure 2H**). We found that downregulation of FOXC2 decreased the tumor volume, tumor weight, and Ki-67 positivity of SGC7901/ADR cells receiving ADR treatment (**Figures 3J, K** and **Supplementary Figure 2J**). Taken together, these results suggest that FENDRR may promote drug resistance in GC cells by performing an enhancer-like role to facilitate FOXC2 expression.

### FENDRR Increases the Expression of FOXC2 by Sponging miR-4700-3p

**Figure 4:**
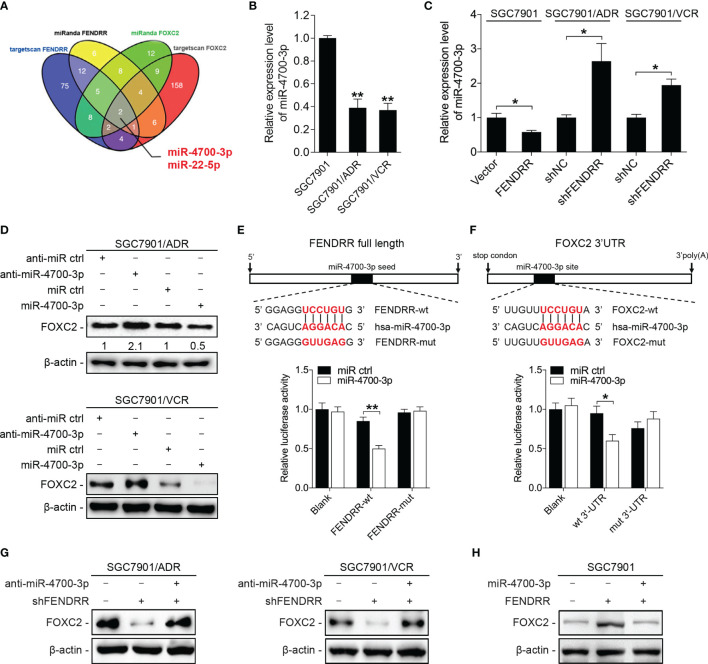
FENDRR increases FOXC2 expression by competitively binding miR-4700-3p **(A)** Prediction of the potential miRNAs targeting both FENDRR and FOXC2 with two independent miRNA target databases. **(B)** qRT-PCR analysis of miR-4700-3p expression in the indicated cells. **(C)** The expression of miR-4700-3p following the downregulation or upregulation of FENDRR expression in the indicated cells. **(D)** Western blot analysis of FOXC2 expression in SGC7901/ADR and SGC7901/VCR cells transfected with miR-4700-3p mimics or inhibitors and the corresponding negative controls. **(E, F)** Predicted potential binding sites of miR-4700-3p to FENDRR and FOXC2 are shown (upper panel). The wild-type (wt) and mutant (mut) miR-4700-3p target sequences of FENDRR **(E)** and FOXC2 **(F)** were fused to a luciferase reporter and cotransfected into HEK-293 cells with miR-4700-3p mimics or a negative control. Luciferase activity values were measured and normalized to empty vector control values. **(G, H)** Western blot analysis of FOXC2 expression in the indicated cells. **P < 0.01, *P < 0.05, *N.S.*, not significant (*P* > 0.05), error bars, s.d.

Cytoplastic lncRNAs can function as ceRNAs by competitively binding specific miRNAs and indirectly regulating the expression of miRNA-targeted genes. As FENDRR was observed to be almost equally located in both the nucleus and the cytoplasm of GC MDR cells (**Figure 3C** and **Supplementary Figure 2D**), we wondered whether FENDRR regulates the expression of FOXC2 through a ceRNA-associated mechanism. According to predictions by multiple algorithms, we identified two miRNAs (miR-4700-3p and miR-22-5p) that could bind to both FENDRR and FOXC2 (**Figure 4A**). miR-4700-3p expression was significantly downregulated in SGC7901/ADR and SGC7901/VCR cells compared with that observed in SGC7901 cells, whereas miR-22-5p exhibited no significant variation in expression (**Figure 4B** and **Supplementary Figure 3A**). Therefore, we focused on miR-4700-3p for further investigation. We observed that silencing FENDRR increased miR-4700-3p expression in MDR cells, while overexpressing FENDRR decreased miR-4700-3p expression in SGC7901 cells (**Figure 4C**). Upregulation of miR-4700-3p reduced FOXC2 protein expression, while downregulation of miR-4700-3p increased FOXC2 protein expression in MDR cells (**Figure 4D** and **Supplementary Figure 3B**). We next examined whether miR-4700-3p can simultaneously bind to FENDRR and FOXC2. Through dual-luciferase reporter assays, we observed that miR-4700-3p overexpression significantly suppressed luciferase activity from the wild-type FENDRR reporter construct but not the construct in which the miR-4700-3p binding site was mutated (**Figure 4E**). Similarly, miR-4700-3p overexpression suppressed the luciferase activities of the FOXC2 3’-UTR reporter constructs, whereas the effect was abolished when the miR-4700-3p binding site was mutated (**Figure 4F**). To further determine whether the FENDRR/miR-4700-3p interaction is functionally involved in the regulation of FOXC2 expression, we co-transfected a miR-4700-3p inhibitor with FENDRR shRNA into MDR cells and observed that miR-4700-3p inhibition abrogated the downregulation of FOXC2 induced by FENDRR knockdown (**Figure 4G**). Consistently, co-transfection of the miR-4700-3p mimic with the FENDRR construct in SGC7901 cells offset the increase in FOXC2 expression induced by FENDRR upregulation (**Figure 4H**). Collectively, these results suggest that FENDRR can modulate FOXC2 expression by sponging miR-4700-3p in GC cells.

### miR-4700-3p Sensitizes GC Cells to Chemotherapy by Targeting FENDRR/FOXC2

**Figure 5:**
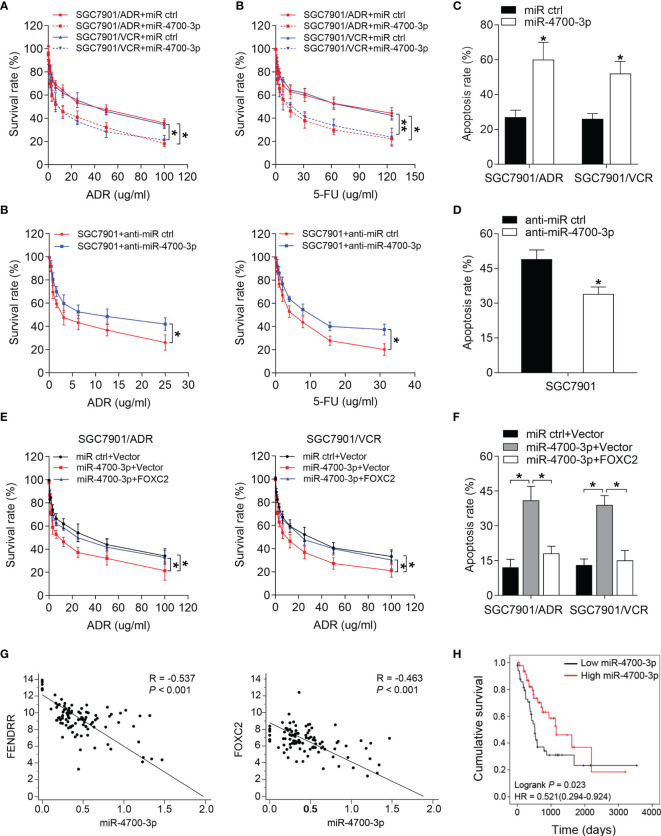
miR-4700-3p suppresses drug resistance in GC **(A, B)** Survival of SGC7901/ADR and SGC7901/VCR cells transfected with miR-4700-3p mimics **(A)** and SGC7901 cells transfected with miR-4700-3p inhibitors or the corresponding negative controls was evaluated using the CCK-8 assay after step-up concentration of ADR and 5-FU treatment for 72 h. **(C, D)** The apoptotic rate of the indicated cells treated with 5-FU was shown. **(E, F)** SGC7901/ADR and SGC7901/VCR cells were cotransfected with miR-4700-3p, FOXC2 vector or their negative controls. Cell survival **(E)** and apoptosis rate **(F)** of the indicated cells treated with ADR or 5-FU, respectively, was evaluated. **(G)** Representative data extracted from TCGA datasets showing correlation between FENDRR (left) and FOXC2 (right) expression with miR-4700-3p expression in GC tissues (n=100). **(H)** Kaplan-Meier analysis of the correlation between miR-4700-3p expression and overall survival in GC patients included in the TCGA datasets (n=100). **P < 0.01, *P < 0.05, error bars, s.d.

Considering the therapeutic potential of miRNAs, we further investigated whether miR-4700-3p could antagonize the FENDRR/FOXC2-mediated MDR in GC. CCK-8 assay results showed that miR-4700-3p overexpression significantly decreased the survival rate of MDR cells treated with ADR and 5-FU, while inhibition of miR-4700-3p produced the opposite effect in SGC7901 cells (**Figures 5A, B**). Overexpression of miR-4700-3p also led to a significant increase in the apoptosis rate of MDR cells, while inhibition of miR-4700-3p reduced the apoptosis induced by chemotherapy in SGC7901 cells (**Figures 5C, D** and **Supplementary Figure 3C**). We then determined whether miR-4700-3p sensitizes MDR cells to chemotherapy by targeting FOXC2. To this end, we co-transfected the miR-4700-3p mimic with a FOXC2 construct lacking its 3’-UTR into MDR cells and observed that FOXC2 co-transfection significantly abrogated the decreased drug resistance induced by ectopic miR-4700-3p expression (**Figures 5E, F** and **Supplementary Figure 3C**), suggesting that FOXC2 is the functional target of miR-4700-3p in the MDR phenotype of GC cells. These results demonstrated the key role of miR-4700-3p in the drug resistance of GC, suggesting that delivery of miR-4700-3p mimics may represent a clinically realistic approach to circumvent the FENDRR/FOXC2-mediated drug resistance in GC. To explore the clinical relevance of our results, we evaluated the association between expression level of FENDRR, FOXC2, and miR-4700-3p using the datasets from the Cancer Genome Atlas (TCGA). We found that there is a significantly negative correlation between FENDRR and FOXC2 with miR-4700-3p expression in 100 cases of paired GC tissues (**Figure 5G**). In addition, low levels of miR-4700-3p expression indicated a poor prognosis of GC patients (**Figure 5H**).

### FOXC2 Expression Is Positively Correlated With FENDRR Expression in GC Tissues

**Figure 6:**
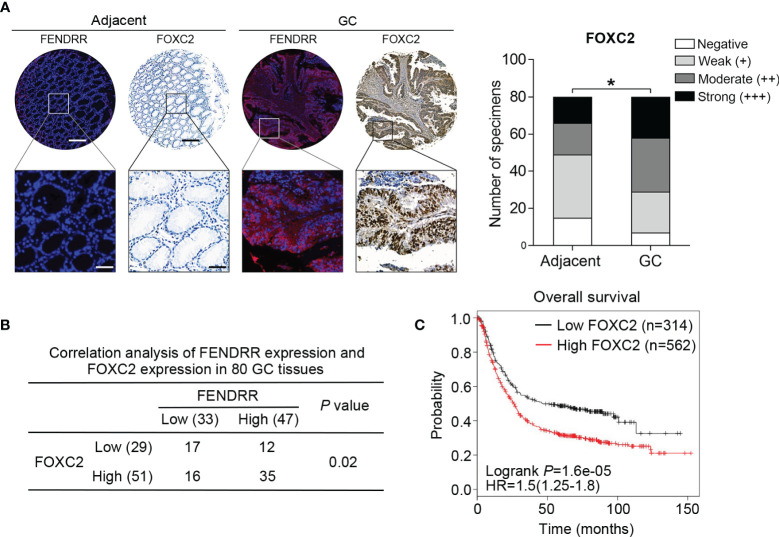
Positive correlation between FENDRR and FOXC2 expression in human GC tissue samples **(A)** Representative images of FENDRR and FOXC2 expression in 80 paired GC and adjacent normal tissue samples detected by FISH and IHC, respectively (left). Scale bar, 200 μm (low magnification) or 50 μm (high magnification). Analysis of immunohistochemical staining for FOXC2 in 80 paired CRC specimens and matched adjacent normal tissue samples (right). **(B)** Correlation between the expression of FENDRR and FOXC2 in 80 GC patients. **(C)** Kaplan-Meier analysis of the correlation between FOXC2 expression and overall survival in patients with GC. *P < 0.05, error bars, s.d.

To elucidate the clinical association of FENDRR and FOXC2 in GC, we examined FOXC2 expression *via* immunohistochemistry (IHC) with the same tissue microarray previously used to detect FENDRR expression (**Figure 1C**). IHC staining results showed that FOXC2 exhibited relatively higher expression in the GC tissue samples than in the corresponding adjacent normal tissue samples, which was consistent with the observed expression pattern of FENDRR (**Figure 6A**). Correlation analysis results also revealed a significant positive correlation between FENDRR and FOXC2 expression in the GC tissue samples (P = 0.02, **Figure 6B**). Moreover, we observed that high FOXC2 in expression GC tissues was significantly associated with more aggressive tumor phenotypes (**Table 2**) and shorter overall survival of GC patients than in patients with lower FOXC2 expression, as confirmed using the Kaplan-Meier plotter database (**Figure 6C**). Taken together, these observations suggest that FOXC2 expression is elevated in GC tissues and that its enhancement is correlated with increased FENDRR.

**Table 2:** Correlation of FOXC2 expression and patients’ clinicopathological variables in GC tissues.

Clinicopathological variables		Tumor FOXC2 expression			*P* Value
		All cases (n = 80)	Low (n = 29)	High (n = 51)	
Age (years)	<50	28	9	19	0.632
	≥50	52	20	32	
Gender	Female	37	14	23	0.819
	Male	43	15	28	
Tumor size	<5 cm	35	19	16	0.005
	≥5 cm	45	10	35	
Grade of differentiation	G1	19	11	8	0.025
	G2	31	12	19	
	G3	30	6	24	
Tumor invasion	T1	13	7	6	0.031
	T2	18	10	8	
	T3	29	9	20	
	T4 a/b	20	3	17	
Lymph node metastasis	Absent	34	18	16	0.01
	Present	46	11	35	
Distant metastasis	M0	31	16	15	0.032
	M1	49	13	36	

## Discussion

Characterization of lncRNA functions requires the elucidation of their molecular mechanisms of action. A growing number of lncRNAs, such as MALAT1, NEAT1, and NKILA, have been shown to function as either tumor suppressors or oncogenes in various cancer types (15). FENDRR has been extensively demonstrated to play tumor suppressive roles in cancer studies. However, paradoxical roles for FENDRR have also been reported (25, 40). In GC, it was reported previously that FENDRR acts as a tumor suppressor by inhibiting the migration and invasion of GC cells by downregulating fibronectin1 expression (41). In the present study, we provided substantial evidence that FENDRR acts as an oncogene involved in GC drug resistance. Firstly, FENDRR overexpression was observed in drug resistant GC cells, PDXs, and clinical tissue samples, and elevated expression of FENDRR was shown to be correlated with poor prognosis in the GC patient cohort. Secondly, we demonstrated that FENDRR can sensitize GC MDR cells to chemotherapy by upregulating the expression of FOXC2, as demonstrated by *in vitro* and *in vivo* experiments. The differences in the phenotype and disease stage studied may account for these discrepancies and contradictions, which strongly support the idea of the complex effects of FENDRR in tumor progression. Indeed, the specific mechanisms underlying FENDRR performing context-dependent functions still need to be investigated further.

FOXC2 is a well-known carcinogenic transcription factor that participates in drug resistance in various cancers (39). However, its role in MDR in GC has not been investigated. In the present study, we provide the first evidence that FOXC2 promotes MDR in GC, observing that FOXC2 expression was elevated in MDR GC cells and GC tissue samples, and its overexpression was correlated with a poor prognosis in GC patients. Importantly, we uncovered dual mechanisms of FOXC2 upregulation in GC. We observed that upregulation or downregulation of FENDRR expression in GC cells led to significant increases and decreases in FOXC2 expression, respectively. A positive correlation was observed between FENDRR and FOXC2 expression in GC tissue microarrays. A potential mechanism for this result may be that FENDRR facilitates activity of TFs or other components necessary for the transcription of FOXC2 by forming a triplex with local DNA double strands, thereby promoting FOXC2 expression. In addition, we reported an alternative mechanism by which FENDRR regulates FOXC2 expression *via* miRNA. Recent studies have demonstrated that miRNAs can be sponged by some lncRNAs located in the cytoplasm, forming a ceRNA network that regulate the expression of target molecules (42). In liver cancer, the KRAL/miR-141/Keap1 network was shown to reverse 5-FU resistance (43). In breast cancer, the NONHSAT101069/miR-129-5p/Twist1 axis was shown to promote Epirubicin resistance (44). In GC, the lncRNA CRAL/miR-505/CYLD/AKT and the D63785/miR-422a/MEF2D axes were demonstrated to regulate CDDP and doxorubicin resistance, respectively (45, 46). In the present study, we showed that miR-4700-3p is functionally involved in FENDRR/FOXC2-mediated MDR in GC. miR-4700-3p sensitized MDR cells to chemotherapy, and FOXC2 was validated as its functional target, which may provide a promising method to antagonize the elevated FENDRR/FOXC2 axis in GC MDR.

In summary, the results of our study identified a potential role of FENDRR in GC MDR and uncovered the underlying mechanisms. We showed that FENDRR overexpression promoted MDR in GC *via* FOXC2 upregulation that was mediated by FENDRR performing an enhancer-like role. In addition, FENDRR was demonstrated to sponge miR-4700-3p, thereby increasing FOXC2 expression indirectly. The FENDRR/miR-4700-3p/FOXC2 network therefore offers novel insight into GC MDR and may represent an effective therapeutic target for GC.

## Data Availability Statement

The raw data supporting the conclusions of this article will be made available by the authors, without undue reservation.

## Ethics Statement

The studies involving human participants were reviewed and approved by Xijing Hospital’s Protection of Human Subjects Committee. The patients/participants provided their written informed consent to participate in this study. The animal study was reviewed and approved by Fourth Military Medical University Animal Care Committee.

## Author Contributions

Conceptualization, XDZ and YN. Data curation, HL, ZZ, and YH. Formal analysis, HL, ZZ, YH, and AF. Investigation, HL and YHL. Methodology, YH. Project administration, ZZ and YN. Resources, XYZ. Software, HML. Supervision, DF, XDZ, and YN. Validation, ZZ and RZ. Visualization, HL, AF, and HML. Writing—original draft, HL and XDZ. Writing—review and editing, YYL, WNL and YN. All authors contributed to the article and approved the submitted version.

## Funding

This study was supported by the National Natural Science Foundation of China (No. 81602641, 81822031, 81871913, 81730016, and 81972761), Young Elite Scientists Sponsorship Program by CAST (2017QNRC001), and Project funding of Xi’an Children’s Hospital (2019D06) and China Postdoctoral Science Foundation.

## Conflict of Interest

The authors declare that the research was conducted in the absence of any commercial or financial relationships that could be construed as a potential conflict of interest.
