# Supplementary figures and images for: The FENDRR/FOXC2 Axis Contributes to Multidrug Resistance in Gastric Cancer and Correlates With Poor Prognosis
Source: Front Oncol. 2021 Mar 22;11:634579. doi: 10.3389/fonc.2021.634579 (PMC8044876; doi:10.3389/fonc.2021.634579)

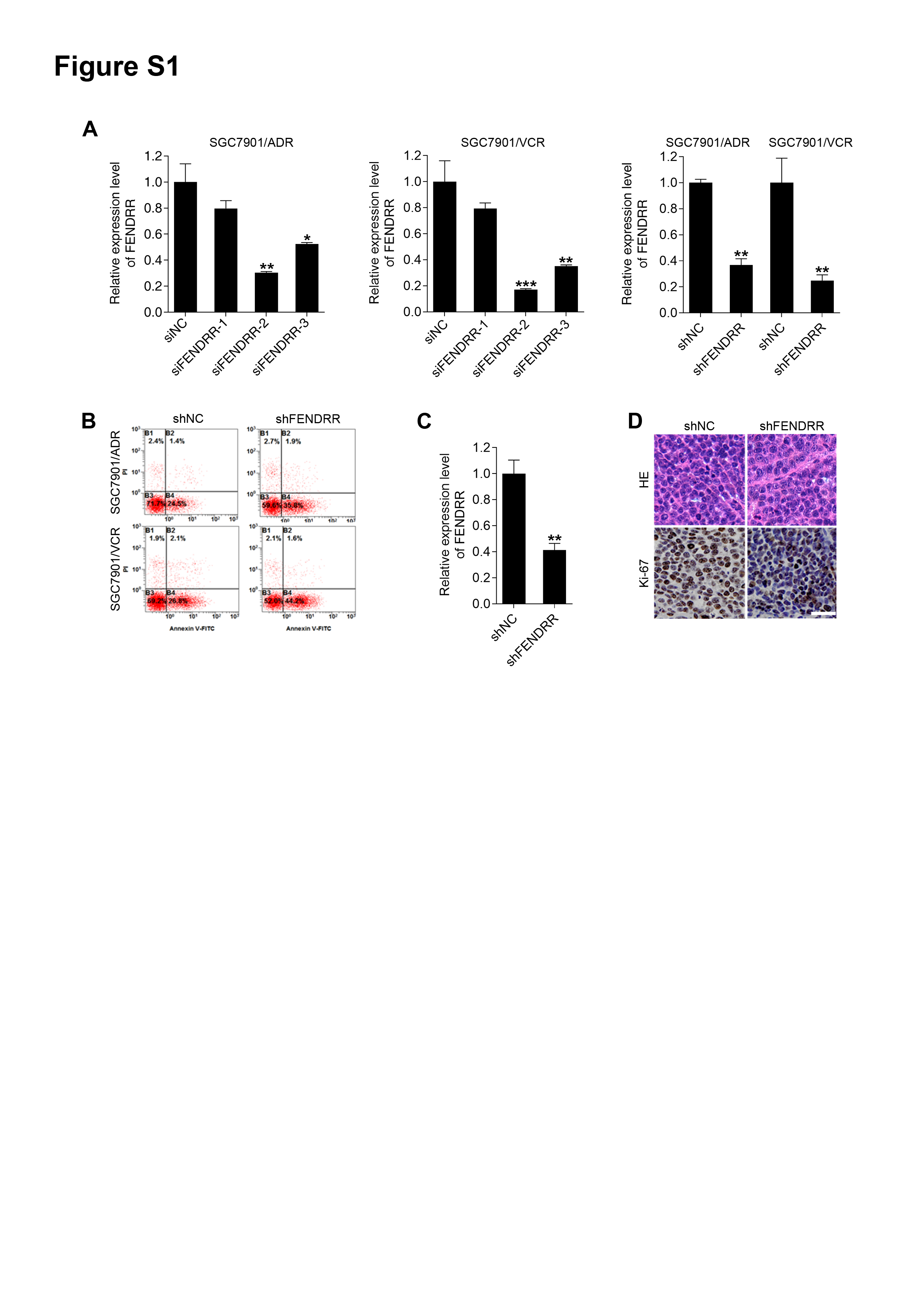

Supplement: Supplementary file 1 [file Image_1.tif]

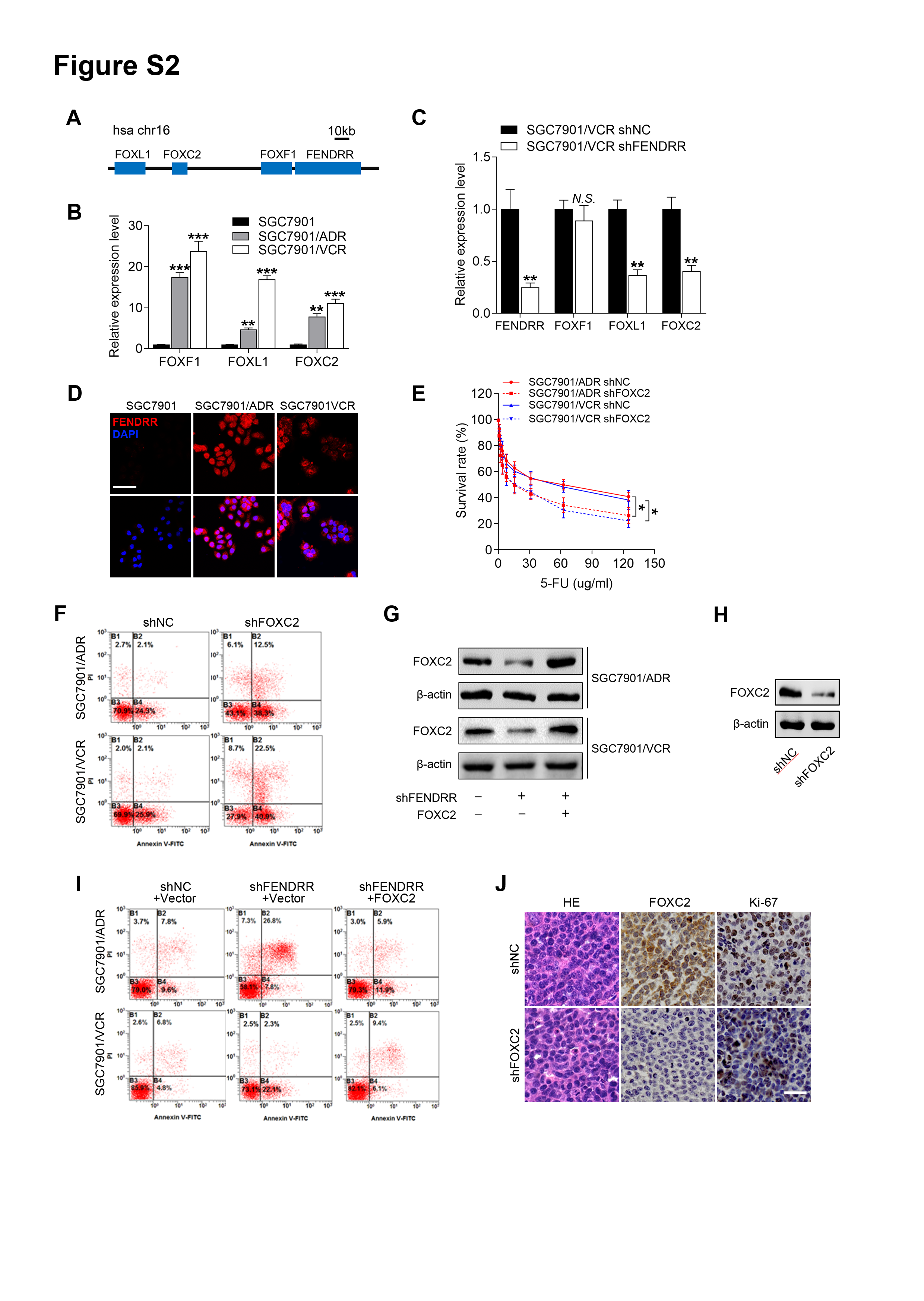

Supplement: Supplementary file 2 [file Image_2.tif]

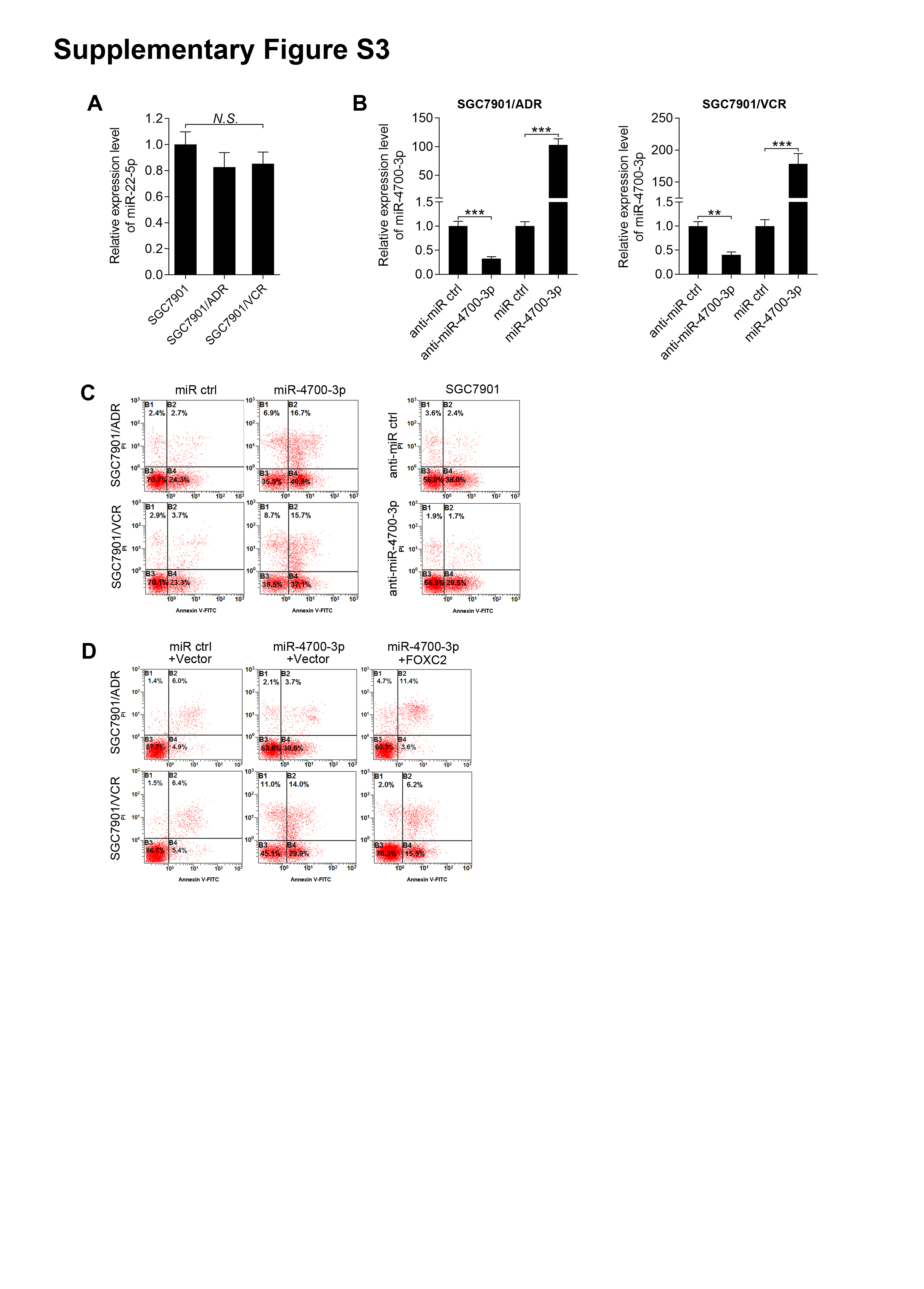

Supplement: Supplementary file 3 [file Image_3.tif]
