# Supplementary material for: The FENDRR/FOXC2 Axis Contributes to Multidrug Resistance in Gastric Cancer and Correlates With Poor Prognosis
Source: Front Oncol. 2021 Mar 22;11:634579. doi: 10.3389/fonc.2021.634579 (PMC8044876; doi:10.3389/fonc.2021.634579)
Supplement: Supplementary file 4 [file Table_1.docx]

**Supplementary Table S1. Primer sequences used in the study**

| Primer name | Primer sequence |
| --- | --- |
| Primers for real-time PCR: |  |
| FENDRR sense: | 5’- TCTGTATCCTTGCCCTGTGG -3’ |
| FENDRR antisense: | 5’- TGGGACATCTGAATCCTGGT -3’ |
| FOXC2 sense: | 5’- CGCCTAAGGACCTGGTGAAG-3’ |
| FOXC2 antisense: | 5’- GGAAGCGGTCCATGATGA -3’ |
| ABCB1 sense: | 5’-GGCTGATTGGCTGGGCAGGAA-3’ |
| ABCB1 antisense: | 5’-TGGAACGGCCACCAAGACGTG-3’ |
| ACTIN sense: | 5’- GGACTTCGAGCAAGAGATGG -3’ |
| ACTIN antisense: | 5’- AGCACTGTGTTGGCGTACAG -3’ |
